# Chemical Constituents from* Daphne giraldii* Nitsche and Their Contents Simultaneous Determination by HPLC

**DOI:** 10.1155/2016/9492368

**Published:** 2016-04-14

**Authors:** Huyiligeqi, Xiaoxv Dong, Chunjing Yang, Guanling Xu, Sali Cao, Jing Fu, Longfei Lin, Jian Ni

**Affiliations:** ^1^Beijing University of Chinese Medicine, Beijing 100029, China; ^2^Affiliated Hospital, Inner Mongolia University for Nationalities, Tongliao 028000, China

## Abstract

*Daphne giraldii* Nitsche (Thymelaeaceae) is widely distributed in the Chinese provinces of Shaanxi, Gansu, and Qinghai, which has been used in Chinese folk medicine to treat ache and rheumatism. Pharmacologic tests have revealed that the plant has anti-inflammatory, analgesic, and anticancer activities. However, there is still not enough systemic investigation on the chemical constituents and the method for the contents simultaneous determination in* D. giraldii*. Therefore, the isolation and characterization of the compounds from the stem barks of this plant were reported. Moreover, a facile, accurate, and reliable method has been developed and validated for their simultaneous determination using HPLC-DAD.

## 1. Introduction

*Daphne giraldii* Nitsche (Thymelaeaceae), commonly called “Zu Shima,” is widely distributed in the northwest areas of China [1, 2]. It has been used in Chinese folk medicine to treat ache and rheumatism, especially for toothache, waist ache, rheumatoid arthritis, and quadriplegia [3–5]. In addition, it has antitumor, analgesic, and anti-inflammatory activities [6]. In the present paper, seven compounds were isolated from the alcoholic extract of the stem bark of* D. giraldii*. Their structures were characterized on the basis of ^1^H and ^13^C NMR spectral data. Currently, there are several reports comprised of analytical methods available for the determination of phenylpropanoids by HPLC. In these reports, several combined techniques with HPLC, such as HPLC-NMR and HPLC-MS, are adopted in the qualitative and quantitative analyses of phenylpropanoids. However, there have not been any reports on the simultaneous determination of the contents of phenylpropanoids in the* D. giraldii*, until now. Thus, the aim of the present investigation was to develop a facile, rapid HPLC method for the analysis of phenylpropanoids in the* D. giraldii* using HPLC-DAD.

## 2. Materials and Methods

### 2.1. Materials and Reagents

The stem barks of* D. giraldii* were provided by the Shanhaiguan Pharmaceutical Factory and identified by Professor Chunsheng Liu (Beijing University of Chinese Medicine). Compounds** 1**–**7** were isolated and identified in our laboratories. All of their purity detected by HPLC was over 99%. HPLC-grade acetonitrile was obtained from Fisher (USA). HPLC-quality water was obtained using a Cascada*™* IX-water Purification System (Pall Co., USA). Other reagents were all of analytical grade. Column chromatography was performed on silica gel H (200–300 mesh, Yantai, China), RP-18 silica gel (ODS, 25–40 *μ*m, Merck), and Sephadex LH-20 (Pharmacia). TLC analysis was run on HSGF 254 precoated silica gel plates (10–40 *μ*m, Yantai, China) and macroporous resin (AB-8, Tianjin, China).

### 2.2. Apparatus

NMR spectra were obtained on a Bruker DRX500 NMR at 500 MHz for ^1^H NMR and 125 MHz for ^13^C NMR. Chemical shifts were reported in ppm with TMS as internal standard. Column chromatography was performed on silica gel H (200–300 mesh, Yantai, China), RP-18 silica gel (ODS, 25–40 *μ*m, Merck), and Sephadex LH-20 (Pharmacia). TLC analysis was run on HSGF 254 precoated silica gel plates (10–40 *μ*m, Yantai, China) and macroporous resin (AB-8, Tianjin, China). All analyses were performed on a Shimadzu HPLC system, equipped with LC-20AT pump, a Shimadzu SCL-10A system controller, and a SPD-20A DAD-UV detector. All separations were carried out on a column Agilent 5 TC-C_18_ column (250 mm × 4.6 mm, 5 *μ*m). The chromatographic data were recorded and processed with a Shimadzu Labsolution workstation.

### 2.3. Chromatographic Conditions

In this system, the mobile phase consisted of acetonitrile (A) and 0.1% (v/v) PA in water (B) and was filtered through a 0.45 *µ*m organic membrane prior to use. The following gradient elution was used: 0–10 min, 95 → 85% B; 10–40 min, 85% B; 40–50 min, 85 → 65% B; 50–70 min, 65% B; 70–80 min, 65 → 5% B; 80–81 min, 5 → 95%; 81–90 min, 95% B. The detected wavelength was 320 nm. The injection volume was 2 *μ*L and the column temperature was maintained at 30°C.

### 2.4. Isolation and Identification of Phenylpropanoids in* D. giraldii*

The dried stem bark of* D. giraldii* was extracted with 70% methanol three times at room temperature, and the solvent was removed under reduced pressure. Subsequently, the crude extract was suspended in water (30 L) and subjected to a macroporous resin AB-8 column eluted with distilled water, 10%, 80%, and 95% ethanol, respectively. The 80% ethanol extract was submitted to column chromatography over silica gel (100–200 mesh) eluting with petroleum ether-EtOAc-methanol (0.5 : 1 : 1 to 1 : 1 : 0.5) to yield fractions 1-2. Fractions 1-2 were subjected to repeated silica gel column chromatography, respectively, eluted with 70% methanol to obtain* giraldoid A* (**1**),* giraldoid B* (**2**),* isolariciresinol* (**6**), and* vladinol D* (**7**). Fractions 3-4, eluted with gradient petroleum ether-EtOAc (20 : 1 to 1 : 1), were further purified by ODS and Sephadex LH-20 column to afford* daphneticin* (**3**),* daphnetin* (**4**), and* pinoresinol* (**5**).

### 2.5. Standard Solution and Sample Preparation

For assay of the four analytes in* D. giraldii*, the standard stock solutions of* giraldoid A* (487 *μ*g/mL),* giraldoid B* (457 *μ*g/mL),* daphneticin* (457 *μ*g/mL), and* daphnetin* (550 *μ*g/mL) were prepared in methanol for each analyte and then diluted with methanol to appropriate concentrations for the establishment of calibration curves. All of the standard solutions were kept at 4°C.

The dried powders of* D. giraldii* samples were accurately weighed and refluxed with 70% methanol (50 mL) for 90 min. Then the resultant mixture was adjusted to the original weight and the supernatant was filtered through a 0.22 *μ*m membrane prior to HPLC injection. The injection volume was 2 *μ*L. All samples were prepared for analysis in triplicate.

### 2.6. Method Validation

#### 2.6.1. Linearity

An aliquot of 10 *μ*L of solution for each calibration standard solution was injected in triplicate for HPLC analysis. The calibration curve was constructed by plotting the peak areas versus the concentration for each analyte. The linearity was evaluated by linear regression analysis and the minimally acceptable correlation coefficient (*r*) was 0.99. The results are shown in Table 1.

#### 2.6.2. Precision and Accuracy

The precision test was determined by performing replicated analysis of the same standard solution six times during the same day and evaluated by the RSD (%) values of the peak area of the analytes. Recovery was used to further evaluate the accuracy of the method. The spiking known quantities of the mixed standard solution were added to known amounts of* D. giraldii* samples. The resultant samples were extracted and then analyzed with the HPLC procedure described above. The content of each phenylpropanoid was determined by the corresponding calibration curve, and the content of each spiked standard was calculated by subtracting the detected amount of the corresponding phenylpropanoid in the control from the total content. Recoveries were calculated by using the ratio of detected amount to that of marker added. The results are shown in Table 2.

#### 2.6.3. Stability

The stability of the analytes was assessed during the sample storing and processing procedure. Short-term stability samples were assessed at room temperature for 24 h. The data are summarized in Table 3.

## 3. Results and Discussion

### 3.1. Structural Elucidation

**Figure 1:**
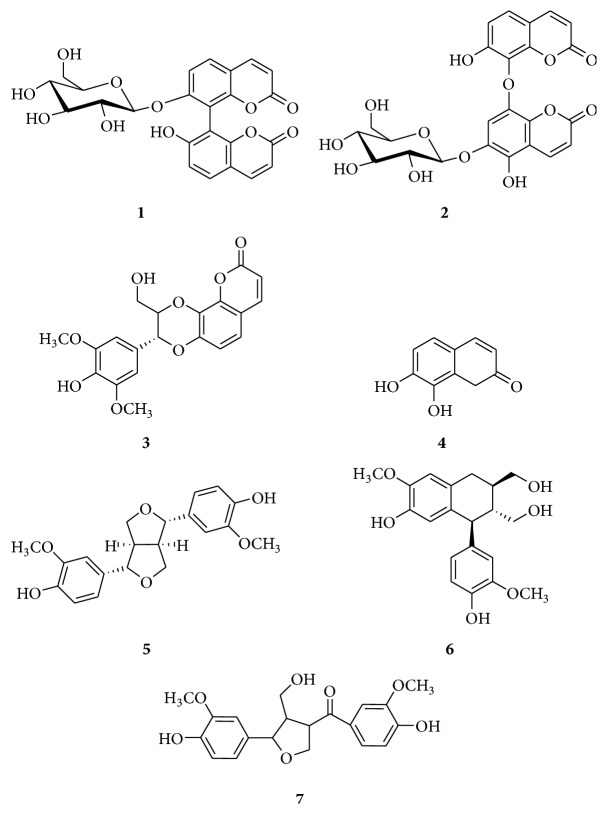
Structures of compounds** 1**–**7**.

*Giraldoid A*. C_24_H_20_O_11_; white amorphous powder; FeCl_3_-K_3_-[Fe(CN)_6_] reaction: positive; ^1^H NMR (CD_3_OD, 500 MHz): *δ*_H_ 7.99 (1H, d, *J* = 10.0 Hz, H-4′), 7.92 (1H, d, *J* = 10.0 Hz, H-4), 7.68 (1H, d, *J* = 10.0 Hz, H-5′), 7.52 (1H, d, *J* = 10.0 Hz, H-5), 7.21 (1H, d, *J* = 10.0 Hz, H-6′), 6.89 (1H, d, *J* = 10.0 Hz, H-6), 6.32 (1H, d, *J* = 10.0 Hz, H-3′), 6.08 (1H, d, *J* = 10.0 Hz, H-3), 4.92 (1H, d, *J* = 10.0 Hz, H-1′′), 2.85 (1H, m, H-2′′), 3.35 (1H, m, H-3′′), 3.12 (1H, m, H-4′′), 3.23 (1H, m, H-5′′), 3.36 (1H, m, H-6′′a), 3.55 (1H, m, H-6′′b); ^13^C NMR (CD_3_OD, 125 MHz): *δ*_C_ 161.9 (C-2), 111.1 (C-3), 146.1 (C-4), 130.8 (C-5), 114.5 (C-6), 160.5 (C-7), 106.0 (C-8), 154.7 (C-9), 115.0 (C-10), 160.9 (C-2′), 114.5 (C-3′), 146.5 (C-4′), 130.8 (C-5′), 112.6 (C-6′), 160.0 (C-7′), 111.1 (C-8′), 154.0 (C-9′), 114.5 (C-10′), 101.9 (C-1′′), 78.6 (C-2′′), 77.9 (C-3′′), 74.7 (C-4′′), 70.9 (C-5′′), 62.0 (C-6′′); the structure of** 1** was shown in Figure 1 [4].

*Giraldoid B*. C_24_H_20_O_13_; white amorphous powder; FeCl_3_-K_3_-[Fe(CN)_6_] reaction: positive; the ^1^H and ^13^C NMR spectra of compound** 2** were similar to those of** 1**; the structure of** 2** was shown in Figure 1 [7].

*Daphneticin*. C_20_H_18_O_8_; yellow powder; FeCl_3_-K_3_-[Fe(CN)_6_] reaction: positive; ^1^H NMR (CD_3_OD, 500 MHz): *δ*_H_ 3.41 (1H, m, H-9′), 3.67 (1H, m, H-9′), 3.66 (6H, s, 2 × OCH_3_), 4.32 (1H, m, H-8′), 4.20 (1H, d, *J* = 8.0 Hz, H-7′), 4.23 (1H, s, 9′-OH), 6.22 (1H, d, *J* = 10.0 Hz, H-3), 6.65 (2H, s, H-2′,6′), 6.86 (1H, d, *J* = 9.0 Hz, H-6), 7.10 (1H, d, *J* = 10.0 Hz, H-5), 7.89 (1H, d, *J* = 10.0 Hz, H-4), 8.45 (1H, s, 4′-OH).^13^C NMR (CD_3_OD, 125 MHz): *δ*_C_ 57.6 (2 × OCH_3_), 61.4 (C-9′), 78.2 (C-7′), 79.5 (C-8′), 106.9 (C-2), 112.9 (C-6), 114.6 (C-3), 114.8 (C-10), 121.3 (C-5), 127.3 (C-1′), 132.5 (C-4′), 137.6 (C-8), 146.3 (C-4), 147.8 (C-7), 149.4 (C-3′), 149.7 (C-9), 161.3 (C-2); the structure of** 3** was shown in Figure 1 [8].

*Daphnetin*. C_9_H_6_O_4_; white powder; FeCl_3_-K_3_-[Fe(CN)_6_] reaction: positive; ^1^H NMR (500 MHz, DMSO-*d*_6_, d): *δ*_H_ 6.20 (1H, d, *J* = 9.0 Hz, H-3), 6.82 (1H, d, *J* = 8.0 Hz, H-6), 7.01 (1H, d, *J* = 8.0 Hz, H-5), 7.90 (1H, d, *J* = 9.0 Hz, H-4), 9.37 (1H, brs, 8-OH), 10.13 (1H, brs, 7-OH). ^13^C NMR (125 MHz, DMSO-*d*_6_, d): 160.4 (C-2), 149.7 (C-7), 145.1 (C-9), 143.8 (C-4), 132.2 (C-8), 118.9 (C-5), 112.5 (C-6), 112.1 (C-10), 111.1 (C-3); the structure of** 4** was shown in Figure 1 [9].

*Pinoresinol*. White amorphous powder; C_22_H_22_O_6_. FeCl_3_-K_3_-[Fe(CN)_6_] reaction: positive; ^1^H NMR (CD_3_OD, 500 MHz): *δ*_H_ 3.15 (2H, s, H-8,8′), 3.70 (6H, s, 2 × OCH_3_), 3.74 (2H, s, H-9a,9′a), 4.29 (2H, s, H-9b,9′b), 4.74 (2H, d, *J* = 4.0 Hz, H-7,7′), 6.72 (2H, d, *J* = 8.0 Hz, H-5,5′), 6.87 (2H, dd, *J* = 8.0, 2.0 Hz, H-6,6′), 6.88 (2H, d, *J* = 2.0 Hz, H-2,2′); ^13^C NMR (CD_3_OD, 125 MHz): *δ*_C_ 54.3 (C-8,8′), 56.0 (2 × OCH_3_), 70.7 (C-9,9′), 87.4 (C-7,7′), 110.7 (C-2,2′), 115.6 (C-5,5′), 119.1 (C-6,6′), 132.8 (C-1,1′), 146.4 (C-4,4′), 147.7 (C-3,3′); the structure of** 5** was shown in Figure 1 [10].

*Isolariciresinol*. White amorphous powder; C_20_H_24_O_6_. FeCl_3_-K_3_-[Fe(CN)_6_] reaction: positive; ^1^H NMR (CD_3_OD, 500 MHz): *δ*_H_ 6.58 (1H, d, *J* = 8.0 Hz, H-5′), 6.49 (1H, s, H-2), 6.39 (1H, d, *J* = 1.5 Hz, H-2′), 6.37 (1H, dd, *J* = 1.5, 8.0 Hz, H-6′), 5.98 (1H, s, H-6), 3.64 (1H, m, H-8′), 1.74 (1H, m, H-8), 2.58 (2H, d, *J* = 10.0 Hz, H-7), 3.32 (1H, dd, *J* = 4.0, 11.0 Hz, H-9′b), 3.46 (1H, m, H-9), 3.62 (2H, m, H-9′a), 3.59 (6H, s, -OCH_3_), 1.51 (1H, m, H-7′); ^13^C NMR (CD_3_OD, 125 MHz): *δ*_C_ 128.6 (C-1), 114.7 (C-2), 148.8 (C-3), 146.1 (C-4), 117.7 (C-5), 134.2 (C-6), 33.8 (C-7), 40.0 (C-8), 65.1 (C-9), 138.6 (C-1), 113.3 (C-2), 147.0 (C-3), 145.6 (C-4), 116.7 (C-5), 122.9 (C-6), 47.4 (C-7), 48.3 (C-8), 61.3 (C-9), 57.1 (-OCH_3_), 57.0 (-OCH_3_); the structure of** 6** was shown in Figure 1 [10].

*Vladinol D*. Colorless needle crystal; C_20_H_22_O_7_. FeCl_3_-K_3_-[Fe(CN)_6_] reaction: positive; ^1^H NMR (CD_3_OD, 500 MHz): *δ*_H_ 7.46 (1H, d, *J* = 1.9 Hz, H-2′), 6.81 (1H, d, *J* = 1.7 Hz, H-2), 6.78 (1H, d, *J* = 7.4 Hz, H-6′), 6.77 (1H, d, *J* = 8.4 Hz, H-5), 6.62 (1H, dd, *J* = 8.1 Hz, H-6), 4.44 (1H, d, *J* = 10.0 Hz, H-7), 4.06 (1H, m, 8-H), 4.03 (1H, t, *J* = 8.4 Hz, H-9′b), 4.00 (1H, dd, *J* = 8.3, 5.5 Hz, H-9′a), 3.72 (3H, s, −OCH_3_), 3.65 (3H, s, −OCH_3_), 3.37 (1H, m, 8-H), 3.66 (1H, m, H-9); ^13^C NMR (CD_3_OD, 125 MHz): *δ*_C_ 199.1 (C-7), 153.4 (C-4), 149.1 (C-3), 148.9 (C-4′), 147.2 (C-3′), 134.0 (C-1′), 129.7 (C-1), 124.9 (C-6), 120.6 (C-6′), 116.5 (C-5), 116.5 (C-5′), 113.1 (C-2), 112.1 (C-2′), 84.4 (C-7′), 71.5 (C-9′), 61.2 (C-9), 57.1 (-OCH_3_), 57.0 (-OCH_3_), 54.8 (C-8′), 50.2 (C-8); the structure of** 7** was shown in Figure 1 [11].

### 3.2. Optimization of Extraction Procedure

Mobile phase systems, extraction solvent, and extraction time were investigated in an effort to optimize the extraction procedure. First, mobile phase systems such as acetonitrile-water, methanol-water, and acetonitrile-water-acetic acid were added to the system. Finally, the acetonitrile-0.1% (v/v) PA system was chosen to obtain better separation and peak shapes. Second, different solvents including 50% methanol, 70% methanol, and 100% methanol were used with heat-refluxing extraction to evaluate the efficiency of the solvent extraction. 70% methanol was the most suitable extraction solvent. Third, the influence of the extraction time on the efficiency of extraction was also optimized. Powdered samples from the* D. giraldii* were extracted with 70% methanol for 30, 60, and 90 min, respectively. Results showed that the samples were extracted for 90 min; the contents of the investigated compounds were satisfactory.

**Figure 2:**
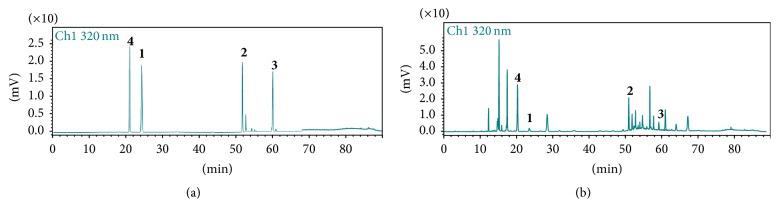
Chromatograms for analysis of compounds** 1**,** 2**,** 3**, and** 4**. (a) Standard solution and (b) sample solution of* D*.* giraldii*. (**1**) Compound** 1**, (**2**) compound** 2**, (**3**) compound** 3**, and (**4**) compound** 4**. The S/N of compounds are 37.61, 404.49, 97.32, and 570.45, respectively.

In the present study, since HPLC with UV detection is a popular method for phenylpropanoids analysis, method development was based on HPLC coupled with DAD. The detection wavelength was set at 320 nm because of the maximum UV absorption wavelengths of the analytes. Under the same chromatographic conditions, chromatographic peaks were identified by comparing their retention times and UV absorption spectra with those of the reference standards. The results showed excellent agreements between the standards and analytes. The chromatograms of standard mixture solution and sample solution of* D. giraldii* were displayed in Figure 2, respectively. Four compounds were found to be well resolved for quantification.

### 3.3. Method Validation

All calibration curves showed good linearity (*r* > 0.99) within a particular concentration range. Typical equations for the calibration were shown in Table 1. The precision test indicated that the RSDs of the peak areas of the analytes were 0.58% (**1**), 0.87% (**2**), 0.43 (**3**), and 0.49% (**4**), respectively, indicating good precision of the developed method. As shown in Table 2, the recovery of the analytes was between 99.2 and 99.8% and the RSDs were less than 1.13%, which indicated good accuracy of the analysis. As shown in Table 3, the RSD values of the peak area for the tests of sample stability were less than 2.91%, indicating that the sample solution was stable when stored at room temperature for 24 h. From the results showing, the method was considered to have good precision, accuracy, and stability.

### 3.4. Sample Analysis

The established HPLC method was subsequently applied to determine the contents of compounds** 1**–**4** in* D*.* giraldii*. Chromatograms are presented in Figure 2. The contents were calculated with the calibration curves method and the data were summarized in Table 4 with the mean values of three parallel determinations.

*D. giraldii* possesses a wide range of pharmaceutical properties, including antitumor, anti-inflammatory, antifertility, and antithrombotic activities. Previous phytochemical research on this plant mainly focused on lignans, diterpenoids [12], coumarins [4], and flavonoids [13]. In present search for natural products with biological activities, three known compounds,* pinoresinol*,* isolariciresinol*, and* vladinol D*, were separated from the* Daphne* genus for the first time; the others were identified as being* giraldoid A*,* giraldoid B*,* daphneticin*, and* daphnetin*, respectively. Among them, compounds** 1**–**4** with high content in crude materials had been used as the chemical marker to evaluate the quality of* D. giraldii* and its related preparations. Hence, in this study, a facile, rapid HPLC method for the contents simultaneous determination in* D. giraldii* was established. However, the activity and the pharmacokinetics of the compounds should be further studied in vivo and in vitro.

## Figures and Tables

**Table 1:** Linear relation between peak area and concentration.

Compound	Regression equation	*r*	Linear range (mg/mL)
**1**	*Y* = 873539*X* + 365.82	0.9996	0.0025–0.05
**2**	*Y* = 146534*X* + 2684.7	0.9998	0.02–0.5
**3**	*Y* = 742641*X* + 1888.6	0.9988	0.005–0.1
**4**	*Y* = 143758*X* + 1024.6	0.9984	0.025–0.55

**Table 2:** Accuracy for the determination of four analytes.

Compounds	Recovery (%)	RSD (%)
**1**	99.2	1.13
**2**	99.3	0.69
**3**	99.7	0.93
**4**	99.8	0.93

**Table 3:** Stability for the determination of four analytes.

Time	The peak area of compounds			
	**1**	**2**	**3**	**4**
0	18127	92613	22037	169176
2	18286	93196	21134	167758
4	18034	91334	20858	167698
8	17859	90760	20691	168257
12	18348	92674	21106	169035
24	18296	92417	22174	169132
RSD (%)	1.03	1.00	2.91	0.41

**Table 4:** Contents of four analytes in *D. giraldii*.

Compounds	Content (mg/g)	RSD (%)
**1**	0.53	1.89
**2**	3.07	0.68
**3**	1.25	1.24
**4**	4.25	1.06
